# Regional Chemotherapy in Locally Advanced Pancreatic Cancer: RECLAP Trial

**DOI:** 10.1186/1745-6215-12-129

**Published:** 2011-05-19

**Authors:** Jeremy L Davis, Prakash Pandalai, R Taylor Ripley, Russell C Langan, Seth M Steinberg, Melissa Walker, Mary Ann Toomey, Elliot Levy, Itzhak Avital

**Affiliations:** 1 Surgery Branch, Center for Cancer Research, National Cancer Institute, NIH, Bethesda, MD 20892, USA; 2 Interventional Radiology Department, Center for Cancer Research, National Cancer Institute, NIH, Bethesda, MD 20892, USA; 3 Biostatistics and Data Management Section, Center for Cancer Research, National Cancer Institute, NIH, Bethesda, MD 20892, USA

## Abstract

**Background:**

Pancreatic cancer is the fourth leading cause of cancer death in the United States. Surgery offers the only chance for cure. However, less than twenty percent of patients are considered operative candidates at the time of diagnosis. A common reason for being classified as unresectable is advanced loco-regional disease.

A review of the literature indicates that almost nine hundred patients with pancreatic cancer have received regional chemotherapy in the last 15 years. Phase I studies have shown regional administration of chemotherapy to be safe. The average reported response rate was approximately 26%. The average 1-year survival was 39%, with an average median survival of 9 months. Of the patients that experienced a radiographic response to therapy, 78 (78/277, 28%) patients underwent exploratory surgery following regional chemotherapy administration; thirty-two (41%) of those patients were amenable to pancreatectomy. None of the studies performed analyses to identify factors predicting response to regional chemotherapy.

Progressive surgical techniques combined with current neoadjuvant chemoradiotherapy strategies have already yielded emerging support for a multimodality approach to treatment of advanced pancreatic cancer.

Intravenous gemcitabine is the current standard treatment of pancreatic cancer. However, >90% of the drug is secreted unchanged affecting toxicity but not the cancer per se. Gemcitabine is converted inside the cell into its active drug form in a rate limiting reaction. We hypothesize that neoadjuvant regional chemotherapy with continuous infusion of gemcitabine will be well tolerated and may improve resectability rates in cases of locally advanced pancreatic cancer.

**Design:**

This is a phase I study designed to evaluate the feasibility and toxicity of super-selective intra-arterial administration of gemcitabine in patients with locally advanced, unresectable pancreatic adenocarcinoma. Patients considered unresectable due to locally advanced pancreatic cancer will receive super-selective arterial infusion of gemcitabine over 24 hours via subcutaneous indwelling port. Three to six patients will be enrolled per dose cohort, with seven cohorts, plus an additional six patients at the maximum tolerated dose; accrual is expected to last 36 months. Secondary objectives will include the determination of progression free and overall survival, as well as the conversion rate from unresectable to potentially resectable pancreatic cancer.

**Trial Registration:**

ClinicalTrials.gov ID: NCT01294358

## Background

In 2010 there were an estimated 43,140 new cases and 36,800 deaths attributed to pancreatic cancer in the United States [1]. Overall, survival is poor, with approximately 23% of patients living 12 months after diagnosis. Overall 5-year survival is approximately 5% at best [2]. Prolonged survival is possible for patients that undergo complete resection and approximates a median of 18 to 20 months in large series, with or without the addition of single-agent chemotherapy [3]. Unfortunately, less than 20% of patients with pancreatic cancer are considered resectable at the time of diagnosis, most often due to locally advanced or metastatic disease. For patients with inoperable pancreatic cancer chemotherapy may prolong survival and improve quality of life, yet it must be considered truly palliative in patients without a surgical treatment option [4].

Since the 1950s, regional administration of chemotherapy has been evaluated in many cancers and in some cases proven an effective therapy for local and regional disease. The pharmacologic rationale for regional drug delivery is to increase drug concentrations at tumor sites and limit systemic drug exposure and its sequelae [5]. In 1958 Creech et al. described the use of regional isolation perfusion with nitrogen mustard compounds in the treatment of 24 patients with a variety of cancers [6]. This report was the first to employ the use of an extracorporeal circuit in the administration of regional chemotherapy. Since that time, the role of regional chemotherapy administration as an adjunctive therapy in patients with locally advanced or regional disease has been well established. Regional administration of chemotherapy is used to treat local-regional and metastatic disease for many cancer histologies. Examples of effective regional therapy include isolated limb perfusion, hyperthermic intraperitoneal chemotherapy, intrathecal, and intravesicular chemotherapy [7-11]. The Surgery Branch of the National Cancer Institute has accumulated significant experience over the years with limb perfusion, peritoneal perfusion, and liver perfusion.

A comprehensive search of the Medline database was performed by the authors to identify all published reports of regional therapy for pancreatic cancer in the English language literature (manuscript in preparation). Medical subject heading (MeSH) terms used included: pancreatic neoplasms; infusions, intra-arterial; chemotherapy, cancer, regional perfusion. Case reports, dose-escalation trials, and studies of adjuvant regional chemotherapy alone were excluded. Reports including multiple gastrointestinal histologies were included only if the patients with pancreatic cancer diagnoses were clearly identified and data amenable to separate analysis. Data collected included the year of publication, size of series, patient demographics, pathologic details including UICC (International Union Against Cancer) stage, type of regional therapy, toxicity and complications, response rate, and survival rate when available. Instances in which institutions published updated patient data or combined analyses, the most recent publications were used.

Twenty-one reports published between 1995 and January 2010, described 895 patients with pancreatic cancer treated with regional chemotherapy. The majority of these studies were small series or sequential, uncontrolled trials. The majority of the patients (>95%) were diagnosed with pancreatic ductal adenocarcinoma. Virtually all patients were described as having locally advanced (stage III) or metastatic cancer (stage IV) at the time of treatment. In over half of reports (11/21) patients were allowed to have undergone prior curative or palliative surgery and, in studies in which it was reported, 11% of patients (59/543) received radiation or chemotherapy prior to receiving regional chemotherapy. One year survival rates approximated those seen with systemic chemotherapy. Unfortunately, the analysis of heterogeneous reports of a non-standardized treatment strategy is limited not only by the inherent bias associated with each study, but also the retrospective nature of any such review. Despite these limitations, a review of the existing literature on an experimental method of treating this lethal disease is necessary for the advancement of future investigation.

The primary endpoints reported in most of the studies described above were tumor response and survival. World Health Organization (WHO) criteria for objective tumor response were used by 71% (15/21) of studies, while 19% (4/21) cited no objective criteria. The average response rate reported was 25.9% (n = 19 studies). The average 1-year survival was 38.9% (n = 10), with an average median survival of 9 months (n = 18). The most commonly reported toxicities were hematologic and gastrointestinal, however 7 studies that reported toxicity did not use standardized reporting criteria. In total, there were 199 cases of grade 1-2, and 47 cases of grade 3-4 gastrointestinal toxicity; 142 cases of grade 1-2 and 70 cases of grade 3-4 hematologic toxicity. At least two studies reported instances of duodenal ulceration while others reported complications including arterial dissection, catheter dislocation, inguinal hematoma, lymphatic fistula, and deep vein thrombosis. Based on radiographic responses to therapy, 78 out of 277 patients (28%) were taken to surgery following regional chemotherapy administration; thirty-two (41%) of those patients were amenable to pancreatectomy or necrosectomy. There were no complete responses to regional chemotherapy reported. No studies performed analyses to identify factors predicting response to regional chemotherapy. Also, no studies compared survival of responding patients versus non-responding patients.

Regional chemotherapy techniques used in these twenty-one studies included arterial infusion and perfusion, with or without hemofiltration. Celiac axis infusion (CAI) was used in a majority of studies (57.1%) whereas selective arterial infusion (SAI; 23.8%) and hypoxic abdominal perfusion (HAP: 28.6%) were used less often. In two studies, HAP and CAI were utilized sequentially. In an attempt to direct blood flow to tumor or pancreas only, three studies utilized selective arterial embolization prior to arterial infusion. Variations to arterial catheterization, including percutaneous versus open surgical approach, appeared to reflect changes in experience or the use of newer technologies over time. A variety of chemotherapeutic agents were used alone or in combination; 5-FU was used most often (57.1%) followed by mitomycin-C (MMC; 47.6%), cisplatinum (CDDP; 38.1%), gemcitabine (23.8%), mitoxantrone (19%), epirubicin and carboplatin (14.3%), methotrexate (4.8%) and melphalan (4.8%). Three studies also included adjuncts to chemotherapy: warfarin, angiotensin-II, and degradable starch microspheres.

### Rationale for gemcitabine

Gemcitabine is a pro-drug that requires intracellular phosphorylation for conversion to the active difluorodeoxycytidine disphosphate (dFdCDP) and triphosphate (dFdCTP) metabolites. dFdCTP competes with dCTP for incorporation into DNA by DNA polymerase; once incorporated into DNA, dFdCTP is resistant to removal within the DNA strand by DNA polymerase resulting in DNA fragmentation and apoptosis. Additionally, dFdCTP competitively inhibits DNA polymerase resulting in a decrease in intracellular dCTP and preferential incorporation of dFdCTP into DNA (referred to as self-potentiation). The pharmacokinetics of dFdCTP are linear; however, the phosphorylation and metabolism of dFdCTP are saturable at dose rates above 10 mg/m^2^/min [12]. This observation suggests that perhaps smaller doses given over longer period of time may potentiate the cytotoxic effect of gemcitabine. Indeed providing proof of this principle, Tempero et al. reported improved median survival (5.0 vs. 8.0 mo; p = 0.013) and 2-yr survival rates (2.2% vs. 18.3%; p = 0.007) when comparing the recommended dose of single agent gemcitabine 1000 mg/m^2 ^given as a 30-minute infusion weekly, compared with a fixed dose rate (FDR) infusion given at 10 mg/m^2^/min (1500 mg/m^2 ^over 150 minutes) [13,14].

Based on the pharmacology of gemcitabine described above, two studies reported on prolonged administration of gemcitabine. Anderson et al. reported on a phase-I study of a 24 hour infusion of gemcitabine in previously untreated patients with inoperable non-small-cell lung cancer. A total of 24 patients were studied. Gemcitabine was administered intravenously as a 24-hour infusion on days 0, 7 and 14 every 28 days. Dose levels were 10, 20, 40, 80, 120, 180, and 210 mg/m^2^/24 hr. The maximum tolerated dose (MTD) was 180 mg/m^2^/24 hr and the dose limiting toxicity (DLT) was neutropenia and lethargy [15]. Rajdev et al. reported on a phase-I trial of gemcitabine administered as a 96-hour continuous intravenous infusion in patients with advanced carcinoma and lymphoma. Gemcitabine was initially given at 1 mg/m^2^/24 hr for 48 hours, then 72 hours and finally 96 hours [16]. The dose was then increased to 2, 4, 6, 10, 15, 20 and 25 mg/m^2^/24 hr for 96 hours. Thirty four patients were treated with a variety of tumors. The MTD was 8 mg/m^2^/24 hr for 96-hour infusions given every 3 weeks and 6 mg/m^2^/24 hr for 96-hour infusions given every 2 weeks. The most common grade 2 or higher toxicity at all dose levels included fever, dyspnea, mucositis hypotension, nausea, vomiting, and fatigue. Neutropenia and thrombocytopenia were uncommon.

In summary, greater than 80% of patients with pancreatic cancer have unresectable primary tumors at the time of diagnosis, most often due to locally advanced disease. This represents a significant proportion of patients with a small chance for meaningful survival at 5-years. The application of local-regional chemotherapy for advanced pancreatic cancer has been posited not only as a method of effectively treating locally advanced disease, but also as a means of transforming a previously unresectable tumor into a resectable one, thereby potentially improving long-term survival. Therefore, a prospective phase I study has been designed to evaluate the safety and efficacy of selective regional infusion of gemcitabine in patients with locally advanced, unresectable pancreatic cancer.

## Methods

The Regional Chemotherapy in Locally Advanced Pancreatic Cancer (RECLAP) Trial was approved by the Institutional Review Board (IRB) of the National Cancer Institute, National Institutes of Health, Bethesda, Maryland, USA.

### Design

**Figure 1:**
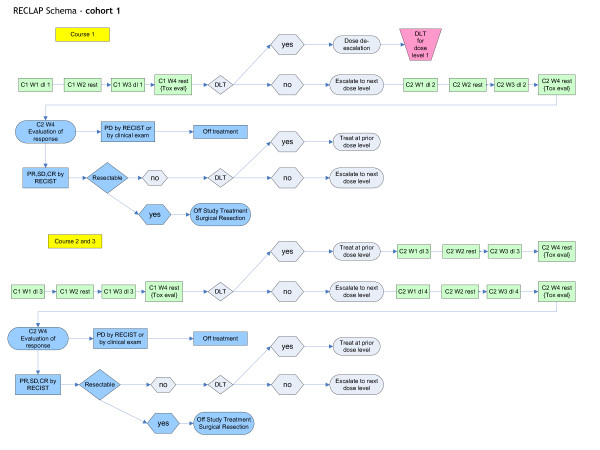
**RECLAP Trial Schema, Cohort 1**. C1 W1 dl1 = Cycle 1, Week 1, dose level 1; C1W3 dl1 = Cycle 1, Week 3, dose level 1, etc.; DLT, dose limiting toxicity; PD, progressive disease; PR, partial response; SD, stable disease; CR, partial response; RECIST, Response Evaluation Criteria in Solid Tumors; Subsequent cohorts will follow the same schema of treatment courses.

This study is a phase I trial with inter-patient and intra-patient dose escalation scheme in which cohorts of patients will be treated with increasing doses of gemcitabine administered as a super-selective continuous arterial infusion over 24 hours. The trial schema is illustrated in Figure 1. The study will be performed at the Clinical Center of the NIH by the Surgery Branch, NCI in Bethesda, Maryland, USA. Patients who have been diagnosed with unresectable, locally advanced pancreatic cancer will be enrolled. The diagnosis must be histologically or cytologically confirmed as pancreatic adenocarcinoma with no extra-pancreatic disease except regional lymph nodes. The disease should be deemed resectable by previously published and accepted criteria for surgical resection [17]. Patients may have previously received chemotherapy and/or radiation therapy.

### Dose-escalation and limiting toxicity

The starting dose of cohort 1 (3 patients) will be 18 mg/m^2^/24 hr, which is 10% of the 24-hour systemic infusion gemcitabine dose. The maximum tolerated dose is the highest dose that induces dose limiting toxicity in no more than 1 patient among a cohort of 6 patients. Patient will be divided into 7 cohorts and will begin dosing at successively higher doses until MTD is established, or up to 165 mg/m^2^/24 hr.

-Cohort 1: 18 mg/m^2^/24 hr

-Cohort 2: 36 mg/m^2^/24 hr

-Cohort 3: 72 mg/m^2^/24 hr

-Cohort 4: 96 mg/m^2^/24 hr

-Cohort 5: 115 mg/m^2^/24 hr

-Cohort 6: 138 mg/m^2^/24 hr

-Cohort 7: 165 mg/m^2^/24 hr

Patients who complete the first cycle of a course without experiencing any dose limiting toxicities may escalate to the next dose level for subsequent cycles. Patients who escalate to higher dose levels will not count towards accrual to the higher cohort but will remain in their initial cohort. The rational for using intra-patient dose escalation is as follows: Unresectable locally advanced pancreatic cancer is a deadly disease without effective therapy; if toxicity is not exhibited by any particular patient, it is reasonable to assume that dose escalation is safe. This will afford patients who start in the early cohorts and do not exhibit toxicity a potential chance to benefit from the trial by progressing to a higher dose level. Toxicity data will be derived from the first dose level and we will analyze the data in terms of cumulative toxicities.

### Data and Safety Monitoring

Careful evaluation to ascertain the toxicity and clinical response will be performed according to the standard of care. Any adverse events will be reported daily, assessed and treated properly by the principal investigator and clinical research team. Adverse events will be graded according to the Common Terminology Criteria for Adverse Events (CTCAE version 4.0). Dose limiting toxicity is defined as all grade 3 or greater toxicities with the exception of grade 3 constitutional symptoms that persist for less than 72 hours, grade 3 and 4 myelosuppression (neutrophils and platelets) of less than 5 days duration, and grade 3 metabolic/laboratory events that are correctable within 24 hours. Accrual will be suspended, the IRB will be notified, and the dose escalation regimen will be reassessed if any of the following criteria are met during the first course (8 weeks) of treatment: Any treatment related deaths within 30 days of treatment, and if 2 of the first 5, or 3 of the first 10, 4 of the first 15, or 5 of the first 20 patients are taken off treatment due to treatment related toxicity. During the post-procedure period patients will receive standard of care supportive measures including analgesics, antiemetics and fluid hydration and all other medically necessary interventions as needed when in the best interest of the patient.

The principal investigator will monitor the data and toxicities to identify trends quarterly, and will be responsible for revising the protocol as needed to maintain safety. The NCI IRB will review submitted adverse events monthly to also evaluate trends and will require a follow up plan from the principal investigator whenever a trend is identified. A Center for Cancer Research (CCR) safety monitoring committee will monitor toxicity trends on this study on at least an annual basis and report any trends to the NCI IRB and principal investigator. Importantly, the study will be continuously monitored by an external monitoring entity that provides study auditing and monitoring services under contract with NIH. The number of patient records monitored is based on actual accrual.

### Statistics

The primary objective of this trial is to determine feasibility, toxicity and tolerability of this treatment after super-selective continuous arterial infusion of gemcitabine in patients with locally-advanced unresectable pancreatic cancer. The secondary objectives include: Conversion rate from unresectable to potentially resectable pancreatic cancer, progression free and overall survival, tumor response rate and to analyze potential selection criteria for patients who present with locally advanced pancreatic cancer that might benefit from this approach. It is expected that a maximum of 15 patients per year can be accrued onto this trial, and thus accrual will be completed in approximately 3 years. Allowing for a very small number of inevaluable patients, the accrual ceiling will be set at 50 patients.

Progression-free survival will also be evaluated using Kaplan-Meier curves and a two-tailed log rank test, as a secondary endpoint. Additionally, a prognostic factor evaluation using Cox proportional hazards modeling will take place after the study has concluded in order to identify factors that are associated with overall or progression-free survival in patients receiving regional chemotherapy; this will be interpreted as a secondary endpoint.

### Inclusion and Exclusion Criteria

#### Inclusion criteria

- Histologically or cytologically confirmed locally advanced pancreatic adenocarcinoma

- Must have evaluable disease by standard radiographic criteria

- Disease should be deemed unresectable

- Patients may be chemo naive or have received prior chemotherapy (including gemcitabine) and/or radiation

- Greater than or equal to 18 years of age

- Must be able to understand and sign the Informed Consent Document

- Clinical performance status of ECOG ≤2

- Life expectancy of greater than three months

- Patients of both genders must be willing to practice birth control during and for four months after receiving chemotherapy

- Absolute neutrophil count greater than 1300/mm^3 ^without the support of filgrastim

- Platelet count greater than 75,000/mm^3^

- Hemoglobin greater than 8.0 g/dl

- Serum ALT/AST less or equal to 3 times the upper limit of normal.

- Serum creatinine less than or equal to 1.8 mg/dl unless the measured creatinine clearance is greater than 60 mL/min/1.73 m^2^

- Total bilirubin less than or equal to 2 mg/dl

- PT within 2 seconds of the upper limit of normal or INR≤1.8

- No history of prior/other malignancies within the 2 years prior to enrollment with the exception of basal cell carcinoma

#### Exclusion criteria

- Metastatic disease including malignant ascites

- Women of child-bearing potential who are pregnant or breastfeeding

- Active systemic infections, coagulation disorders or other major medical illnesses of the cardiovascular, respiratory or immune system, myocardial infarction, heart failure

- Childs B or C cirrhosis or with evidence of severe portal hypertension by history, endoscopy, or radiologic studies

- Weight less than 40 kg

- Significant ascites, greater than 1000cc in the absence of peritoneal disease

- Concomitant medical problems that would place the patient at an unacceptable risk for the procedure

- Need for concurrent chemotherapy

### Intervention

Catheter placement will be performed in the Interventional Radiology Section. The catheterization procedure will be performed as per routine and according to a three-step strategy; 1) arterial redistribution will be performed as necessary, 2) percutaneous placement of the indwelling infusion catheter and port, and 3) evaluation of catheter position and patency and management of drug distribution.

The purpose of arterial redistribution procedures is to 1) reduce pancreatic arterial supply from multiple branches to a single (or few) feeding artery(s) for infusion purposes, and 2) occlude non-target arteries that arise from the chemoinfusion site which supply non-target intraabdominal organs. In general, pancreatic head neoplasms will be supplied by the anterior and posterior pancreaticoduodenal arteries and inferior pancreaticoduodenal arteries, whereas the pancreatic body and cauda tumors are supplied by the dorsal pancreatic artery, the great pancreatic artery, and the caudal pancreatic artery, all branches of the splenic artery. Homma et al. developed a strategy in which pancreatic arterial branches are super-selectively embolized, leaving the great and caudal pancreatic arteries alone arising from the splenic artery as the chemoinfusion source for any tumor in the entire pancreas [18].

Following selective catheterization of the superior mesenteric artery, superior mesenteric arteriography will be performed to identify accessory or replaced hepatic vasculature, as well as to assess the patency of the inferior pancreaticoduodenal arcade. If the pancreaticoduodenal branches from the gastroduodenal artery are stenotic or encased by tumor, coil embolization of the inferior pancreaticoduodenal artery/arcade will be performed through the origin of this vessel from the superior mesenteric artery.

Celiac arteriography will then be performed to identify hepatic and splenic arterial anatomy, followed by selective gastroduodenal and splenic arteriography to recognize pancreatic supply as well as non-target vessels supplying the duodenum, or stomach. The anterior and posterior superior pancreaticoduodenal arteries and the gastroduodenal arteries will then undergo coil embolization. If computed tomography (CT) angiography or similar study previously demonstrated supply to the target pancreatic tumor from the dorsal pancreatic artery (splenic artery origin), then this vessel will be embolized as well. As a result of the embolization steps, the only remaining arterial supply to the pancreatic tumor will be the great pancreatic and caudal pancreatic artery branches of the splenic artery [19]. The above described maneuvers will be performed; however it may not always be possible to perform in all patients due to vascular variation. In these cases a selective infusion will be done via the most prominent artery supplying the tumor.

Following the selective embolization steps described above, the tip of the infusion catheter will be placed in the splenic artery proximal to the great and caudal pancreatic arteries. If a different diagnostic catheter is used for the selective angiography and embolization, catheter exchange will be performed for the infusion catheter in routine fashion. Cone bead CT arteriography will be performed with iodinated contrast injection through the infusion catheter to confirm opacification of the entire pancreas. A subcutaneous pocket will be created in the lower abdominal or lateral thigh soft tissues, and the trailing end of the infusion catheter will be pulled subcutaneously into the pocket for port attachment.

### Post-procedure care and follow up

Patients will be admitted to the NIH Clinical Center and receive routine post-procedure care. Imaging studies will confirm catheter placement the evening of and the morning following the procedure. Initial treatment may begin the morning following embolization or when all procedure related toxicities have resolved and imaging confirms placement of the catheter. During treatment patients will be monitored for pancreatic-related complications and any other gastrointestinal, metabolic, hematologic and constitutional symptoms. Prior to discharge patients will receive instructions regarding subcutaneous port and catheter care, as well as completion of the patient diary and potential toxicities.

Patients will undergo evaluation for response at the end of each course (every 8 weeks) with computed tomography of the chest, abdomen and pelvis with pancreas protocol. Magnetic resonance imaging and positron emission tomography will be used if indicated. Response to therapy will be determined using RECIST criteria and EASL [20,21]. Potential conversion to resectability following treatment will be determined using standard radiographic criteria. Patients must be off chemotherapy for at least 4 weeks and all toxicities must resolve to grade 1 or less prior to surgery.

### Endpoints

#### Primary Objectives

- To evaluate feasibility and toxicity of intra-arterial gemcitabine therapy

- To establish the maximum tolerated dose

#### Secondary Objectives

- To evaluate response rate using RECIST and EASL criteria

- To determine progression free and overall survival

- To evaluate the conversion rate from unresectable to potentially resectable pancreatic cancer

- To analyze potential selection criteria to be used in future studies for patients who present with marginally unresectable or unresectable locally-advanced pancreatic cancer

## Discussion

The optimal treatment for pancreatic cancer is complete resection followed by adjuvant systemic chemotherapy. Due to late-stage diagnosis most patients have limited chance for curative resection, thus novel approaches for early detection and effective treatment must be explored. In addition, new approaches that will convert unresectable locally advanced pancreatic cancer into a resectable state might result in better outcome as surgical extirpation provides the only chance to survive 5 years. In cases which tumors are detected early enough to allow resection, the choice of adjuvant chemotherapy is based on the results of a randomized clinical trial that demonstrated significant improvement in median overall survival favoring gemcitabine over observation alone [3]. The addition of targeted molecular agents or cytotoxic drugs to gemcitabine adds little or no clinical benefit to patients with this disease to date [22,23]. Likewise, although encouraging, current data are not definitive regarding the benefit of adjuvant chemoradiotherapy. Therefore, increasing the rate of resection for patients with pancreatic cancer may represent a practical approach to improve survival for patients currently without a surgical treatment option. Achieving this goal requires neoadjuvant therapy that mediates substantial tumor regression, potentially allowing for complete resection in previously unresectable patients.

This study offers an innovative approach for locally advanced unresectable pancreatic cancer: a twenty-four-hour highly selective intra-arterial infusion of gemcitabine. This approach offers two advantages: First, based on the pharmacodynamics and pharmacokinetics of gemcitabine, a lower dose infused over prolonged time will optimally saturate the enzyme responsible for the conversion of gemcitabine into its two active metabolites resulting in higher concentrations of intracellular active gemcitabine, and second, selective intra-arterial delivery of gemcitabine will avoid systemic toxicity and first pass degradation of gemcitabine by the small-bowel and the liver. Local administration of gemcitabine to the pancreas has been shown to be safe with low morbidity.

## List of abbreviations

**CAI**: celiac artery infusion - the infusion of chemotherapy via an intra-arterial catheter positioned within the celiac artery; **SAI**: selective arterial infusion - the infusion of chemotherapy via an intra-arterial catheter positioned within vessels selected for tumor-specific infusion; **HAP**: hypoxic abdominal perfusion - the perfusion of chemotherapy utilizing arterial inflow catheters, venous return catheters and an extra-corporeal circuit; **MTD**: maximum tolerated dose - the highest dose that induces dose limiting toxicity in no more than 1 patient among a cohort of 6 patients; **DLT**: dose limiting toxicity - defined as all grade 3 or greater toxicities with the exception of grade 3 constitutional symptoms that persist for less than 72 hours, grade 3 and 4 myelosuppression (neutrophils and platelets) of less than 5 days duration, and grade 3 metabolic/laboratory events that are correctable within 24 hours; **ECOG**: Eastern Cooperative Oncology Group; **AST**: aspartate aminotransferase; **ALT**: alanine aminotransferase; **PT/INR**: prothrombin time/international normalized ratio; **RECIST**: Response Evaluation Criteria In Solid Tumors; **EASL**: European Association for the Study of the Liver

## Competing interests

The authors declare that they have no competing interests.

## Authors' contributions

IA is the principal investigator for the study described in the manuscript. JLD and IA are responsible for the concept and design. JLD, PP, RTR, RCL, SMS, MW, MAT, EBL and IA made significant contributions to protocol validity, design, drafting, and revising of the manuscript. SMS developed the statistical considerations for the trial. JLD, PP, RTR, RCL, SMS, MAT, and IA contributed to the scientific accuracy of the manuscript. All authors read and approved the final manuscript.
